# Amplitude-modulated stimuli reveal auditory-visual interactions in brain activity and brain connectivity

**DOI:** 10.3389/fpsyg.2015.01440

**Published:** 2015-10-02

**Authors:** Mark Laing, Adrian Rees, Quoc C. Vuong

**Affiliations:** Institute of Neuroscience, Newcastle University, Newcastle upon TyneUK

**Keywords:** auditory-visual integration, temporal congruence, brain network, psychophysiological interaction, amplitude modulation

## Abstract

The temporal congruence between auditory and visual signals coming from the same source can be a powerful means by which the brain integrates information from different senses. To investigate how the brain uses temporal information to integrate auditory and visual information from continuous yet unfamiliar stimuli, we used amplitude-modulated tones and size-modulated shapes with which we could manipulate the temporal congruence between the sensory signals. These signals were independently modulated at a slow or a fast rate. Participants were presented with auditory-only, visual-only, or auditory-visual (AV) trials in the fMRI scanner. On AV trials, the auditory and visual signal could have the same (AV congruent) or different modulation rates (AV incongruent). Using psychophysiological interaction analyses, we found that auditory regions showed increased functional connectivity predominantly with frontal regions for AV incongruent relative to AV congruent stimuli. We further found that superior temporal regions, shown previously to integrate auditory and visual signals, showed increased connectivity with frontal and parietal regions for the same contrast. Our findings provide evidence that both activity in a network of brain regions and their connectivity are important for AV integration, and help to bridge the gap between transient and familiar AV stimuli used in previous studies.

## Introduction

Everyday events and objects concurrently stimulate multiple senses, and an important task for the brain is to determine whether signals received by different modalities belong to the same or different sources. Perceptually combining different sensory signals from the same source can enhance performance, particularly when environmental conditions are not ideal. For example, visual information about a speaker’s lips can enhance the intelligibility of her spoken speech in a noisy room (Sumby and Pollack, 1954; Grant and Seitz, 2000). Combining information from different sources can lead to multi-sensory illusions; most notably, when the syllable conveyed by a speaker’s voice does not match the one conveyed by her lips, observers perceive a syllable that is neither the auditory syllable nor the visual syllable (McGurk and MacDonald, 1976). There is accumulating behavioral and neural evidence that the strength of multi-sensory integration depends on the congruence between sensory signals. This congruence can be defined by spatial or temporal information, such as sensory signals originating from the same spatial location or occurring in close temporal proximity (e.g., Frassinetti et al., 2002). Congruence can also be defined by semantic information, such as a dog’s bark matching a picture of a dog rather than a picture of a cat (e.g., Naumer et al., 2011).

In the current study, we focused on how temporal congruence facilitates auditory-visual (AV) integration at the neural level. Events in the environment are dynamic and present multi-sensory information continuously over a range of time scales. With many events occurring at similar locations, the temporal congruence of multi-sensory information may be a powerful cue for combining sensory signals: congruence will generally be higher for sensory signals originating from the same source than from different sources. Indeed, temporal congruence can lead to behavioral advantages across various stimuli and tasks. Following the example above, focusing on the speaker’s lips would enhance the intelligibility of her speech despite other simultaneous conversions and events. In this case, the temporal congruence is produced by the synchrony between the continuously changing shape of the lips and the changing amplitude of the speech envelope over an extended period (Grant and Seitz, 2000; Vander Wyk et al., 2010). Not only will the synchrony between the speaker’s lips and speech be higher than between the lips and other environmental sounds, there may also be congruent semantic information derived from lip reading and the speech itself (Calvert et al., 2000). For non-meaningful stimuli (e.g., simple tones and visual shapes), temporal congruence can lead to higher target detection (e.g., Frassinetti et al., 2002; Lovelace et al., 2003; Maddox et al., 2015), better motion discrimination (e.g., Lewis and Noppeney, 2010; Ogawa and Macaluso, 2013) and faster responses (e.g., Nozawa et al., 1994; Diedrich and Colonius, 2004) when the auditory and visual signals are congruent.

Complementing behavioral evidence, human brain imaging studies have identified regions that respond more to AV stimuli than to auditory or visual stimuli alone (e.g., Calvert et al., 1999, 2000, 2001; Beauchamp et al., 2004; Stevenson et al., 2007; Sadaghiani et al., 2009; Stevenson and James, 2009; Vander Wyk et al., 2010; Naumer et al., 2011; for a review see Stein and Stanford, 2008). These putative multi-sensory regions include those within the temporal [e.g., superior temporal sulcus (STS)], the parietal [e.g., intraparietal sulcus (IPS)] and the frontal lobes [e.g., inferior frontal gyrus (IFG)], as well as subcortical structures such as the superior colliculus (Meredith and Stein, 1983). Several of these studies show the importance of temporal congruence in increasing regional activity for congruent AV stimuli and decreasing regional activity for incongruent AV stimuli. In an early human-imaging paper, Calvert et al. (2001) presented auditory white noise bursts in parallel with a visual checkerboard pattern with reversing black and white squares. Each sensory stimulus type had a different duration (auditory: 39 s on, 39 s off; visual: 30 s on, 30 s off) giving rise over time to auditory, visual, and AV periods. In separate blocks, Calvert et al. (2001) also manipulated whether the onset of the sound and onset of the checkerboard occurred at the same time (congruent) or whether the onsets were randomly out of temporal phase with respect to each other (incongruent). Observers listened passively to all stimuli. Importantly, their study showed that temporal congruence led to response enhancement when the auditory and visual signals were congruent and to response suppression when they were incongruent, emphasizing the importance of temporal information for modulating brain activations. Using a similar paradigm, but with speech stimuli, Calvert et al. (2000) found that the temporal congruence of meaningful stimuli also elicited similar response enhancement and suppression, with the strongest response in the left posterior STS. In this study, they paired visual lip movements with either the correct sound track (congruent) or another sound track (incongruent). On incongruent blocks, the mis-match between the lip movements and sound track gave rise to different temporal patterns of the auditory and visual signals (as well as semantic incongruency due to lip reading). These overall patterns of results have been replicated with different types of auditory and visual stimuli such as non-meaningful transient tone-bursts (i.e., “beeps”) and flashes (Noesselt et al., 2007), speech-like stimuli (circles and ellipses animated with speakers’ speech envelopes; Vander Wyk et al., 2010) and meaningful non-speech stimuli (e.g., videos of tool use; Stevenson et al., 2007; Stevenson and James, 2009; Werner and Noppeney, 2010). These studies suggest that congruent AV stimuli typically lead to stronger responses than incongruent AV stimuli but this is not always the case (e.g., Noesselt et al., 2012). For instance, when congruency is defined along a semantic dimension, semantically incongruent AV stimuli can lead to larger responses than semantically congruent AV stimuli (e.g., Hocking and Price, 2008; Meyer et al., 2011; Beer et al., 2013).

The regional responses to AV stimuli are important but they do not necessarily provide a complete picture of multi-sensory integration at the neural level for at least two complementary reasons. First, there are anatomical connections between brain regions, allowing information to be transmitted quickly between them (Felleman and Van Essen, 1991; Beer et al., 2011, 2013; van den Brink et al., 2014). Second, brain regions can show functional connectivity with each other; that is, activity in different regions can co-vary over time (Hagmann et al., 2008). These anatomical and functional connections may, for instance, allow regions to pool information from other regions (e.g., Noppeney et al., 2010; Beer et al., 2013). Several human studies have investigated brain connectivity patterns for AV integration (e.g., Noesselt et al., 2007, 2012; Lewis and Noppeney, 2010; Noppeney et al., 2010; Werner and Noppeney, 2010; Lee and Noppeney, 2011; Ogawa and Macaluso, 2013; Kim et al., 2015). For example, Werner and Noppeney (2010) found interactions between auditory and visual regions (see also Lewis and Noppeney, 2010, and Ogawa and Macaluso, 2013, for motion discrimination). They had observers categorize videos of everyday actions as tools or instruments, and varied both the presence of a sensory signal and (if present) how informative it was about the action. Auditory and visual signals were degraded by adding visual or auditory noise. This manipulation reduced the reliability of the sensory signal, which is known to increase the strength of multi-sensory integration. The concurrent presentation of a visual signal automatically increased responses in auditory cortex via direct connectivity with the visual cortex or indirectly through the STS. Interestingly, Noesselt et al. (2012) found that perceived temporal congruence could also modulate functional connectivity. They presented observers with AV speech streams in which the auditory stream was physically leading, the visual stream was physically leading, or the streams were physically synchronous. The authors further manipulated the stimulus onset asynchrony between the auditory and visual streams to create bistable percepts. That is, observers would perceive physically asynchronous AV streams (visual leading or auditory leading) sometimes as asynchronous and sometimes as synchronous. Noesselt et al. (2012) found that despite the same physical stimuli (e.g., visual leading), there was an increased functional connectivity between the STS and right prefrontal regions when observers correctly perceived the AV stimulus as asynchronous relative to when they incorrectly perceived the AV stimulus as synchronous. For transient auditory tone and visual flash stimuli, Noesselt et al. (2007) found increased functional connectivity between the STS and primary visual and auditory regions, rather than frontal regions, when the tones and flashes were temporally coincident (synchronous) relative to when they were temporally non-coincident (asynchronous).

Most human imaging studies have focused on speech, music and other meaningful (e.g., animals or tools) stimuli that carry high-level cognitive and/or semantic information. We do not know if the same brain regions are activated by simpler AV constructs. Furthermore observers may have differential experiences with familiar stimuli, which can shape how the brain responds to them. For example, Lee and Noppeney’s (2011) data showed that connectivity could change with expertise. On the other hand, previous studies of AV interactions using non-meaningful AV stimuli often use transient sounds and visual patterns that rarely occur in nature (Sekuler et al., 1997; Shams et al., 2000; Calvert et al., 2001; Noesselt et al., 2007). Here we used continuous sounds and shapes which are nonetheless unfamiliar AV stimuli. These consisted of a three-dimensional object that was sinusoidally modulated in size and combined with a tone that was sinusoidally modulated in amplitude. Both the auditory and visual signals were thus continuous and were modulated at modulation rates commonly experienced in familiar stimuli such as speech (e.g., Plomp, 1983; Rosen, 1992; Shannon et al., 1995). Using these AV stimuli, we reported that observers’ sensitivity to amplitude differences between two sequentially presented AV stimuli were affected if the auditory and visual signals were modulated at the same rate (congruent) but not when they were modulated at different rates (incongruent; Vuong et al., 2014). This temporal manipulation allowed us to test how combining auditory and visual information changes brain activation and/or brain connectivity, without the confound of speech, language, and semantic information. We found that temporally congruent AV stimuli led to increased activation in putative multi-sensory areas in temporal and parietal lobes, consistent with previous reports (e.g., Calvert et al., 2000, 2001; Noesselt et al., 2007, 2012), but temporally incongruent AV stimuli led to increased functional connectivity between auditory/visual regions and predominantly frontal regions (see also Noesselt et al., 2012). Overall, the results suggest that both brain activation and connectivity changes support AV integration. Our results provide an important link between transient, unfamiliar stimuli and continuous real-world objects, speech and music.

## Materials and Methods

### Participants

Nine right-handed adults (seven males, two females; age in years: *M* = 24, *SD* = 1.6; range: 21–26 years) participated in the study. All participants reported normal hearing and normal or corrected-to-normal vision. All participants provided informed consent. The study was performed in accordance with the Declaration of Helsinki and approved by the Ethics Committee of Newcastle University.

### Apparatus

The visual stimuli were back-projected onto a screen at the foot end of the scanner using a canon XEED LCD projector (1280 × 1024 pixels, 60 Hz). Participants viewed the projection through an angled mirror attached to the head coil ~10 cm above their eyes. The sounds were presented using an MR-compatible audio system and delivered with electrostatic transducer headphones (NordicNeuroLab). Participants wore earplugs to further protect against scanner noise. Head motion was restricted by placing foam pads between the head and the head coil. The experiment was run on a Windows 7 PC using the Psychophysics Toolbox version 3^1^ (Brainard, 1997; Pelli, 1997; Kleiner et al., 2007; run on 32-bit MATLAB 2012, Mathworks, Inc.) to control the experiment, present the stimuli and record behavioral responses. Participants responded via a MR-compatible response pad (LumiTouch).

### Stimuli

**FIGURE 1:**
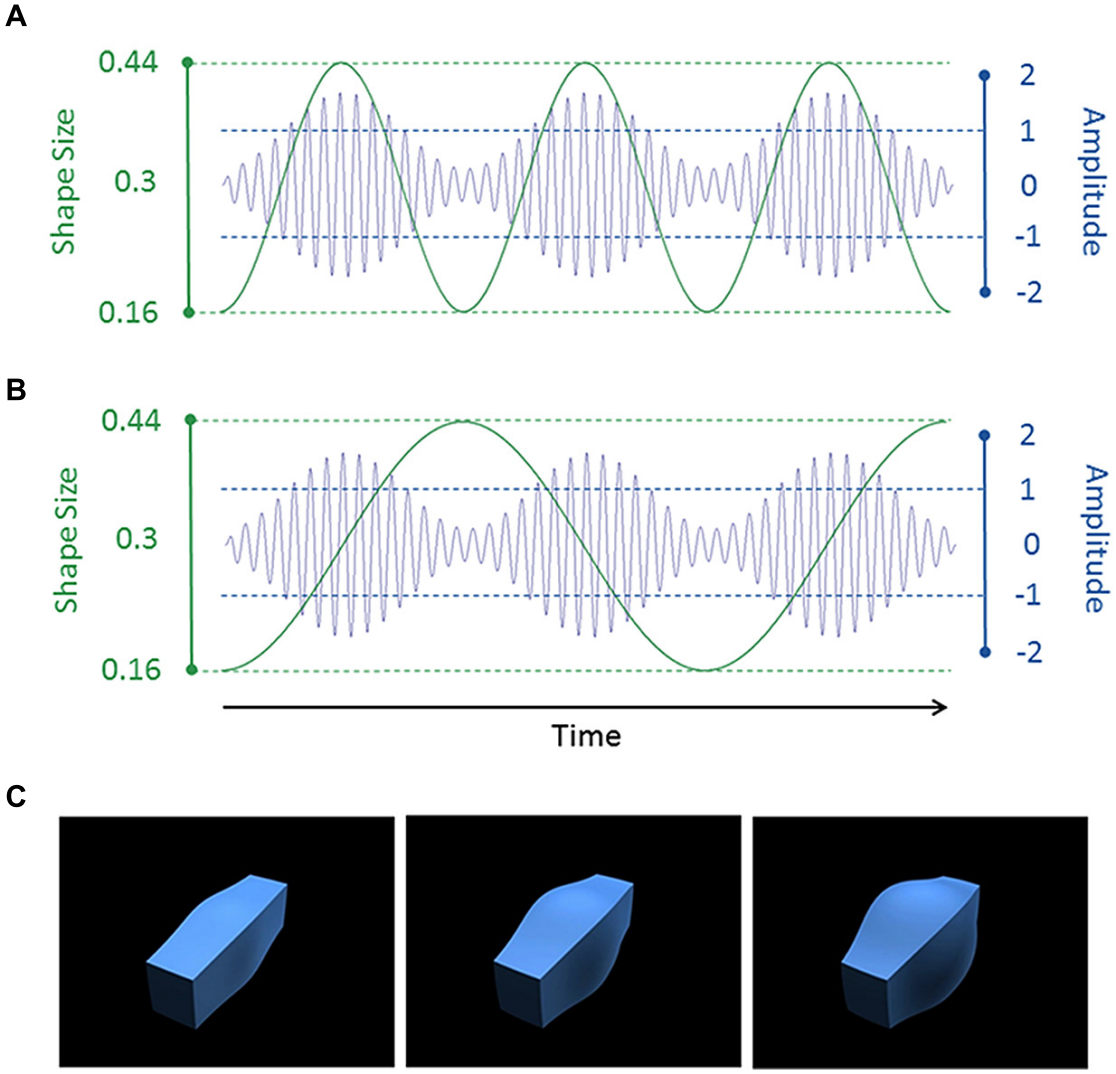
**(A,B)** Example auditory (blue) and visual (green) stimulus waveforms. In **(A)**, the shape size and sound amplitude were modulated at 2 Hz, leading to an AV congruent condition. In **(B)**, the shape size was modulated at 1 Hz and the sound amplitude was modulated at 2 Hz, leading to an AV incongruent condition. The auditory stimulus had a carrier frequency of 250 Hz. For display purposes only, we show a lower frequency of 30 Hz in the figure. **(C)** The shape with a small (0.16, left), medium (0.3, middle) or large (0.44, right) value of the spherify modifier.

**Figure 1** illustrates the auditory and visual stimuli used in the study. The auditory stimuli consisted of amplitude-modulated tones (see **Figures 1A,B**), with a 250 Hz carrier frequency sinusoidally amplitude-modulated at 1 or 2 Hz with a modulation depth of 70%. They were created in MATLAB 2012 and saved as stereo wav files with a 44.1 kHz sampling rate. We were unable to measure the volume within the scanner. We therefore set the sound level of our stimuli to 75 dB SPL in a sound-attenuated room. The sounds were presented via headphones on a high fidelity MR-compatible audio system (NordicNeuroLab). We used a fixed setting for the audio system (volume level = 4) for all participants in the scanner but they could all clearly hear the tones with our sparse imaging protocol.

The visual stimuli consisted of size-modulated three-dimensional (3D) cuboids (see **Figures 1A–C**). The cuboid was created using 3D Studio Max version 7 (Autodesk, Inc.). The “spherify” modifier was applied to a blue rectangular box (1.0 × 1.2 × 4.0 units [width × height × length]) to vary the size of the central portion of the cuboid. This modifier can vary from 0 (rectangle) to 1.0 (sphere). As with the tones, a 1 or 2 Hz sinusoid waveform was used to modulate the modifier between 0.16 and 0.44 (oscillating around a mean of 0.3). The cuboid was rendered against a uniform black background from an oblique camera viewpoint. The bounding box of the cuboid subtended a visual angle of 13.7° × 13.7° (300 pixels × 300 pixels). The videos were saved as Quicktime movie files (240 frames; 60 frames per second; H.264 compression).

The auditory and visual stimuli were 4.0 s in duration. There were thus four cycles with the 1 Hz modulation rate and eight cycles with the 2 Hz rate. The two modalities and two modulation rates were factorially combined to produce four stimuli. Importantly, there were two congruency conditions which reflected whether the auditory and visual stimuli had the same (congruent) or different (incongruent) modulation rates. The 1 Hz modulation rate was considered to be “slow” and the 2 Hz modulation rate was considered to be “fast.”

### Design and Procedure

There were six experimental conditions in the current study. Participants were instructed to attend to either the auditory or visual stimulus. For each attended stimulus, they were presented with the audio- or video-only stimulus (A or V), the AV congruent stimulus (AVC) and AV incongruent stimulus (AVI). Each experimental condition was presented twice in each functional run in a random order giving a total of 12 experimental blocks. Before each experimental block, there was an instruction block to inform participants to attend to the auditory or visual stimulus. Each functional run was ~10 min in duration. There were three functional runs for eight of the participants and two runs for one participant.

A 10.0 s instruction screen appeared before each experimental block in which the label “AUDITION” or “VISION” was presented at the center of the screen (Courier, 64 font size, white text). There were four trials in each 40.0 s experimental block. Participants judged whether the attended stimulus (audio or video) was “slow” (1 Hz) or “fast” (2 Hz) while ignoring the modulation rate of the unattended stimulus (if present). They used a response pad to make their response (with the response mapping counterbalanced across participants). In each 10.0 s trial, a fixation cross was presented for 2.0 s, followed by the stimuli for 4.0 s, and by a blank screen for 2.5 s. Participants could only respond during a 1.5 s period in which the word “respond” was displayed (Courier, 24 font size, white text). If they responded before this period or did not respond within this period, the next trial continued and the response was counted as an error. The fMRI image acquisition occurred at the beginning of each trial whilst the fixation cross was displayed and recorded the brain response to the preceding trial. Thus there was no interference from the scanner noise during the presentation of the auditory stimuli. Outside the scanner, participants were given a practice block for each experimental condition to familiarize them with the trial sequence and enable them to appreciate the difference between “slow” and “fast” auditory and visual stimuli. The modulation-rate judgment task ensured that participants remained alert in the scanner but was designed to be an easy task, and was not used to assess the extent to which participants integrated the AV stimuli.

### Image Acquisition

All participants were scanned at the Newcastle Magnetic Resonance Centre. Anatomical T1-weighted images and functional T2*-weighted echo planar images (EPIs) were acquired from a 3 T Philips Intera Achieva MR scanner using a Philips 8-channel receive-only head coil. The high resolution T1-weighted scan consisted of 150 slices and took approximately 5 min to acquire. The parameters of the structural scan were: repetition time (TR) = 9.6 ms, echo time (TE) = 4.6 ms, flip angle = 8°. The field of view (FOV) was 240 mm × 240 mm × 180 mm with a matrix size of 208 × 208 pixels. Each voxel was 0.94 mm × 0.94 mm × 1.2 mm in size. The T2*-weighted EPIs consisted of 28 axial slices acquired from the bottom to the top of the head. The parameters of the EPIs were: acquisition time (TA) = 1.3 s, TR = 10 s, TE = 30 ms, flip angle = 90°. The FOV was 192 mm × 192 mm × 125.5 mm with a matrix size of 64 × 64 pixels. Each voxel was 3 mm × 3 mm × 4 mm in size, with a 0.5 mm gap between slices. We used sensitivity encoding (SENSE) with factor = 2 to increase the signal-to-noise ratio of the functional images. For each participant, a total of 62 functional images were acquired in each run (~10 min per run). Due to some technical problems, 64 functional images were acquired in each run for one participant. Before each functional run, four “dummy” scans were acquired to allow for equilibration of the T1 signal.

### fMRI Pre-processing

Functional images were realigned to the first image across all runs for each participant and re-sliced to correct for head motion. These images were normalized to a standard Montreal Neurological Institute (MNI) EPI T2*-weighted template with a resampled voxel size of 3 mm × 3 mm × 3 mm. They were then spatially smoothed with a 6 mm full-width-at-half-maximum Gaussian kernel to improve the signal-to-noise ratio and to allow for comparisons across participants. To remove low-frequency drifts in the signal, we applied a high-pass filter with a cutoff of 180 s.

### fMRI Whole-brain Analysis

The preprocessed data were analyzed using SPM8^2^ (Friston et al., 1994). We used the general linear model (GLM) with a two-step mixed-effects approach. First, a fixed-effects model was used to analyze each participant’s data set. Second, a random-effects model was used to analyze the individual datasets at the group level. No additional smoothing of the images was used at the group level.

The design matrix for each participant was constructed as follows. The onset and duration for each of the six experimental blocks and the instruction (baseline) block were modeled as boxcar functions (40.0 s for experimental blocks, 10.0 s for the instruction block). These boxcar functions were convolved with a finite impulse response function (Order 1) implemented in SPM8. In addition to these regressors of interest, the six movement parameters (roll, yaw, pitch, and three translation terms) and a constant term for each session were included in the design matrix as regressors of no interest. A linear combination of the regressors was fitted to the BOLD signal to estimate the beta weight for each regressor.

For the first-level analysis, contrast images were computed from the beta-weight images. We used the contrasts A > instruction and V > instruction to localize uni-sensory auditory and visual regions. There are several statistical criteria for localizing multi-sensory regions (Beauchamp, 2005). Given our temporal congruency manipulation, we focused on the contrast AVC > AVI (averaging across the attention conditions) to localize multi-sensory regions (e.g., Calvert et al., 2000, 2001; Beauchamp et al., 2004; Noesselt et al., 2007, 2012). For the second-level group analysis, one-sample *t*-tests of participants’ contrast images were conducted at each voxel.

The goal of the whole-brain analyses was to functionally localize well-established uni- and multi-sensory regions. These regions served as seeds for the functional connectivity analyses described below. We therefore used a liberal statistical threshold (uncorrected *p* < 0.001 at the voxel level) and we focused on those clusters that were within cortical regions reported in previous studies (e.g., Calvert et al., 2000, 2001; Beauchamp et al., 2004; Noesselt et al., 2007, 2012). For all other statistical tests, we used α = 0.05 and considered 0.05 < *p* < 0.10 as marginal effects.

### fMRI Psychophysiological Interaction Analysis

We used the generalized form of context-dependent psychophysiological interaction (PPI) analyses^3^ (McLaren et al., 2012; see also Friston et al., 1997; Gitelman et al., 2003) to identify regions which show changes in functional connectivity as a function of audio-visual congruency. For the PPI analyses, we derived three regressors from the BOLD time series. First, a regressor representing the physiological activity in a seed area was computed by deconvolving the first eigenvariate of the BOLD time series from all voxels in that area to estimate changes in neural activity in that area. Second, a regressor representing the psychological context was computed by convolving a boxcar time series for the two congruency conditions with the canonical hemodynamic response function implemented in SPM8. To test for increased connectivity on AV congruent trials, AVC blocks were coded as +1 and AVI blocks were coded as -1. Conversely to test for increased connectivity on AV incongruent trials, AVC blocks were coded as -1 and AVI blocks were coded as +1. Lastly and importantly, a regressor representing a PPI was computed by multiplying the first two regressors. These three regressors were used to augment each participant’s design matrix from the whole-brain analyses (see above). In this augmented design matrix, the experimental conditions and head-movement parameters were treated as regressors of no interest to factor out the contribution of the experimental conditions on the PPI analyses (McLaren et al., 2012).

We used the functionally localized uni-sensory and multi-sensory regions (see analysis above) as the bases of our seeds. To generate seed areas, we defined a 6 mm sphere centered on the peak voxel of a given region (i.e., the voxel with the largest response). Only significant voxels within this sphere were included in the seed. Although our multi-sensory regions were based on contrasting AVC and AVI conditions, it is important to note that the PPI regressor combined with factoring out the contribution of the experimental conditions meant that we did not bias our sampling for the multi-sensory seeds. As with the whole-brain analyses, we first estimated regressor beta weights for each participant (first-level analysis). We then submitted the participants’ beta-weight image for the PPI regressor to a one-sample *t*-test against zero for the contrasts AVC > AVI or AVI > AVC (second-level analysis).

## Results

### Behavioral Results

**Table 1** presents the behavioral results in the scanner. As expected, participants had no difficulty distinguishing the fast and slow rates in the modulation-rate judgment task (accuracy > 90%). The proportion correct data and response times from correct trials were submitted to a 2 attended stimulus (audio, video) × 3 AV congruency (audio/video-only, AV congruent, AV incongruent) repeated measures analysis of variance (ANOVA). For accuracy, there was only a marginally significant main effect of attended stimulus, *F*(1,8) = 5.3, *p* = 0.051, $\eta_{p}^{2}$ = 0.40. Participants were marginally more accurate when attending to the visual compared to the auditory stimulus (vision: *M* = 0.97, *SEM* = 0.01; audition: *M* = 0.93, *SEM* = 0.02). The effect of congruency and the interaction between the two factors were not significant, *F*s < 1.0. For correct response times, there was no main effect of attended stimulus or congruency, and there was no interaction between the two factors, all *F*s < 1.4 and *p*s > 0.28.

**Table 1:** Behavioral results in the scanner.

	Attend audio			Attend video		
	Audio	Cong	Incong	Video	Cong	Incong
Proportion Correct (sem)	0.92 (0.05)	0.96 (0.01)	0.92 (0.03)	0.97 (0.02)	0.98 (0.01)	0.97 (0.01)
Correct response time in msec (sem)	548 (34)	554 (32)	570 (26)	578 (31)	568 (25)	564 (23)

### fMRI Whole-brain Results

#### Uni-Sensory Regions

**FIGURE 2:**
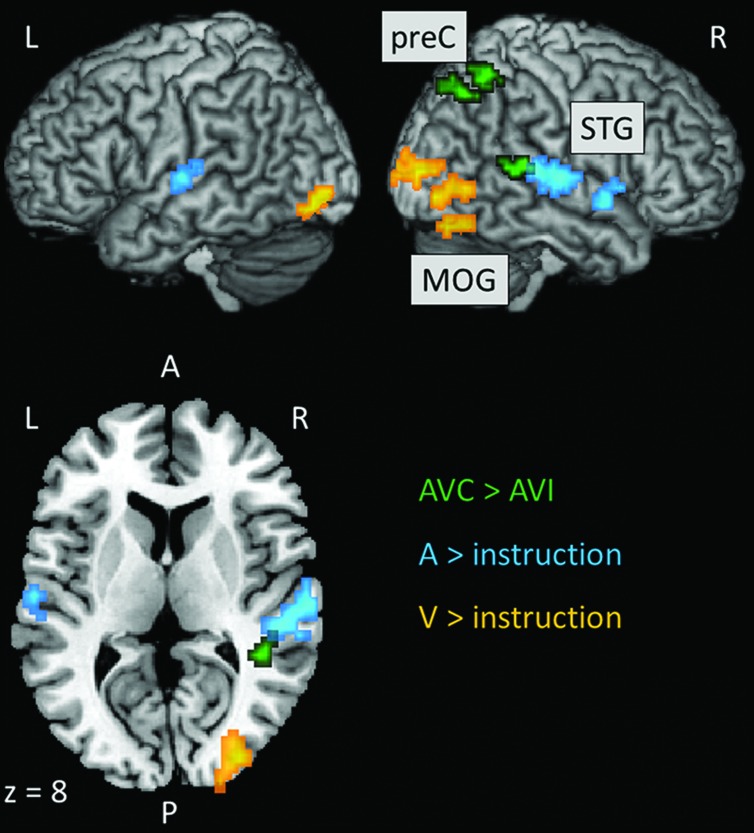
**Results of the whole-brain analyses to localize uni-sensory and multi-sensory regions that were used as the bases for the seeds subsequently used in the PPI analyses.** Slice numbers are in MNI coordinates. L, left; R, right; A, anterior; P, posterior; preC, precuneus; STG, superior temporal gyrus; MOG, middle occipital gyrus.

We localized auditory and visual regions using the contrasts A > instruction and V > instruction, respectively. For the auditory contrast, we used an initial threshold of *p* = 0.01 and *k* = 20. For the visual contrast, we used an initial threshold *p* = 0.001 and *k* = 20. **Tables 2** and **3** present the auditory and visual results, respectively. For these and subsequent tables, we also present regions which had uncorrected *p* < 0.001 peak voxels and we used the WFU Pickatlas toolbox to label the reported regions (with exceptions as noted). The labels are based on the peak voxel (Maldjian et al., 2003). For the auditory contrast, we found activations in the area of the posterior right STG corresponding to Heschl’s gyrus, and activations in the left posterior and right anterior STG. These auditory regions were used as seeds in the PPI analyses below. There were further activations in a white-matter region of the temporal lobe, in frontal regions and in the cerebellum. These clusters are not known to process auditory information. We therefore did not use them as seeds. For the visual contrast, we found activations in the visual cortex (three clusters in the right MOG and one in the left FG). These visual regions were used as seeds in the PPI analyses below. There was a further activation in the medial frontal gyrus, which is not known to process visual information. We therefore did not use this cluster as a seed. **Figure 2** illustrates the auditory and visual regions from the whole-brain analysis that were used as the bases for the seeds used in the PPI analyses.

**Table 2:** Audio-only > instruction results.

Structure	Hem	*k*	*Z*	MNI coordinate			*p_unc_*	*p*_corr_
				*x*	*y*	*z*		
Superior temporal gyrus†	R	80	3.59	51	-31	10	0.0002	0.050
Superior temporal gyrus†	R	21	3.86	54	2	-8	0.0001	0.550
Superior temporal gyrus†	L	25	4.27	-60	-16	7	<10^-5^	0.503
Temporal lobe (sub-gyral)	R	121	4.18	36	-55	-2	<10^-5^	0.010
Medial frontal gyrus	L	258	4.31	-3	5	49	<10^-5^	<10^-5^
Postcentral gyrus	L	429	3.75	-39	-31	61	0.0001	<10^-5^
Cerebellum	R	65	3.45	9	-55	-17	0.0003	0.083

*The † indicates regions used as seeds for the psychophysiological interaction analyses. *k*, cluster size; Z, Z-score; p_*unc*_, uncorrected *p*-value; *p*_*corr*_, cluster-corrected p-value.*

**Table 3:** Video-only > instruction results.

Structure	Hem	*k*	*Z*	MNI coordinate			*p_unc_*	*p*_corr_
				*x*	*y*	*z*		
Middle occipital gyrus†	R	58	4.62	33	-88	7	<10^-5^	<10^-5^
Middle occipital gyrus†	R	50	4.32	45	-82	-5	<10^-5^	<10^-5^
Inferior occipital gyrus†	R	22	3.98	42	-76	-20	<10^-5^	0.017
Fusiform gyrus†	L	29	3.84	-42	-82	-8	0.0001	0.006
Medial frontal gyrus	L	20	4.59	-3	8	49	<10^-5^	0.020

*The † indicates regions used as seeds for the psychophysiological interaction analyses. *k*, cluster size; *Z*, *Z-*score; p_*unc*_, uncorrected *p*-value; *p*_*corr*_, cluster-corrected *p*-value.*

#### Multi-sensory Regions

Following previous studies (Calvert et al., 2000, 2001; Beauchamp et al., 2004; Noesselt et al., 2007, 2012), we used congruency contrasts to localize multi-sensory regions. For these contrasts, we used an initial threshold of *p* = 0.001 and *k* = 20. **Table 4** presents the activations from the AVC > AVI contrast. We found one cluster in the right posterior STG and two clusters in the right parietal lobe that showed activations, which have previously been established as AV regions (e.g., Calvert et al., 2000, 2001; Beauchamp et al., 2004; Noesselt et al., 2007, 2012). We therefore used these clusters as seeds in the PPI analyses below. There was also activation in the left cingulum but this region was not used as a seed as no previous studies reported this region to be involved in processing AV stimuli. **Figure 2** also illustrates the multi-sensory regions from the whole-brain analysis used as the bases for the seeds in the PPI analyses. There were no activations with the AVI > AVC contrast.

**Table 4:** Auditory-visual congruent > Auditory-visual incongruent results (pooling over attention conditions).

Structure	Hem	*k*	*Z*	MNI coordinate			*p_unc_*	*p*_corr_
				*x*	*y*	*z*		
Superior temporal gyrus†	R	20	4.09	42	-37	13	<10^-5^	0.068
Precuneus†	R	20	4.21	9	-67	43	<10^-5^	0.068
Intraparietal sulcus†^1^	R	26	4.21	27	-58	52	<10^-5^	0.068
Cingulum	L	21	3.89	-15	-40	46	0.0001	0.068

**FIGURE 3:**
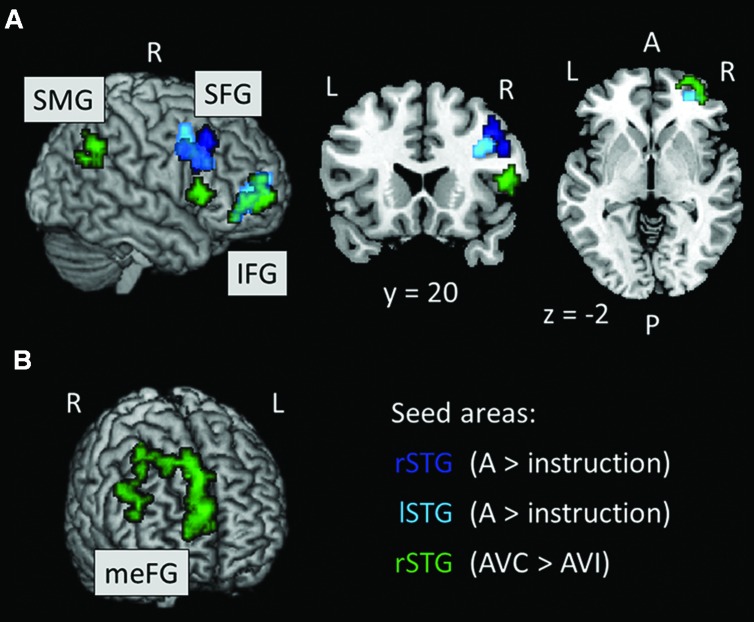
**(A, B)** Results of the PPI analysis for the AVI > AVC contrast. Seed areas refer to areas activated in the whole-brain analyses (**Tables 4** and **5**; **Figure 2**). Slice numbers are in MNI coordinates. L, left; R, right. Note: For display purposes, the large target region in the meFG (*k* = 361) is presented separately in **(B)**. IFG, inferior frontal gyrus; meFG, medial frontal gyrus; SFG, superior frontal gyrus; SMG, supramarginal gyrus.

*The † indicates regions used as seeds for the psychophysiological interaction analyses. *k*, cluster size; Z, *Z*-score; p_*unc*_, uncorrected *p*-value; *p*_*corr*_, cluster-corrected *p*-value. ^1^Although the peak voxel was located in the precuneus, most of the region was in the parietal lobe (see **Figure 3**).*

### fMRI PPI Results

We ran PPI analyses to test whether the functional connectivity between regions depended on whether the AV stimuli were temporally congruent (same modulation rate) or incongruent (different modulation rates). We used uni-sensory and multi-sensory regions identified in the whole-brain analyses to derive our seeds (see regions with † in **Tables 2–4**). For these contrasts, we used an initial threshold *p* = 0.005 and *k* = 20. As shown in **Table 5**, the analyses identified several target regions that showed a positive change in functional connectivity with the different seeds on incongruent relative to congruent AV blocks (i.e., for the contrast AVI > AVC). **Figure 3** illustrates those target regions that were significant at the cluster-corrected level. These regions clustered in frontal and parietal cortices. There was one marginally significant target region in the STG that showed a marginally significant positive change in the functional connectivity with the right auditory seed on congruent relative to incongruent AV blocks. None of the visually localized seeds and none of the significant regions outside of the temporal lobe from the AVC > AVI contrast showed changes in functional connectivity as a function of the temporal congruence between the auditory and visual signals.

**Table 5:** Psychophysiological interaction results.

Structure	Hem	*k*	*Z*	MNI coordinate			*p_unc_*	*p*_corr_
				*x*	*y*	*z*		
**AVI > AVC**					
*Auditory seed* (left superior temporal gyrus, -60 -16 7, *k*_seed_ = 15)					
Inferior frontal gyrus	R	80	3.87	36	20	28	0.0001	0.014
Inferior frontal gyrus	R	29	3.45	51	26	10	0.0003	0.215
Middle frontal gyrus	R	65	3.47	36	44	-8	0.0003	0.022
Middle frontal gyrus	R	26	3.38	27	32	43	0.0004	0.220
Precentral gyrus	R	29	3.47	27	-19	73	0.0003	0.215
Precuneus	R	28	3.36	12	-49	37	0.0004	0.215
Cerebellum	L	22	4.08	-45	-49	-29	<10^-5^	0.290
*Auditory seed* (right superior temporal gyrus, 51 -31 10, *k*_seed_ = 18)					
Middle frontal gyrus	R	105	3.86	39	26	40	0.0001	0.001
Middle frontal gyrus	R	28	3.04	39	14	49	0.001	0.222
Superior frontal gyrus	R	28	3.37	24	44	34	0.0004	0.222
*Congruency seed* (right superior temporal gyrus, 42 -37 13, *k*_seed_ = 7)					
Inferior frontal gyrus	R	46	3.83	51	20	10	0.0001	0.063
Medial frontal gyrus	R	361	3.92	3	32	43	<10^-5^	<10^-5^
Superior frontal gyrus	R	99	3.64	24	62	4	0.0001	0.002
Supramarginal gyrus	R	54	3.79	48	-49	37	0.0001	0.043
**AVC > AVI**					
*Auditory seed* (right superior temporal gyrus, 51 -31 10, *k*_seed_ = 18)					
Superior temporal gyrus	L	52	3.87	-39	-58	16	0.0001	0.08

*k, Cluster size; *Z*, *Z*-score; p_*unc*_, uncorrected *p*-value; *p*_*corr*_, cluster-corrected *p*-value; *k*_*seed*_, number of voxels in the seed.*

## Discussion

We used unfamiliar stimuli to investigate the role of temporal congruence in AV integration and to reveal the underlying neural mechanisms supporting integration. We manipulated temporal congruence by modulating the amplitude of a tone and the size of a 3D cuboid either at the same (congruent) or different (incongruent) amplitude-modulation rate. Here we show that both regional activations in the temporal lobe and functional connectivity between temporal, parietal and frontal regions support AV integration of continuous and unfamiliar stimuli independently of their semantic content.

Using whole-brain analyses, we localized a significant auditory region in the right temporal lobe and significant visual regions in the occipital-temporal lobe. We further found increased activation in the right STG and the right parietal cortex when the modulation rates of the auditory and visual stimuli were temporally congruent (e.g., both modulated at 2 Hz) relative to when they were incongruent (e.g., amplitude modulation at 1 Hz and size modulation at 2 Hz). Although these multi-sensory regions are marginally significant at the cluster level (*p* = 0.068), they are consistent with a large number of previous human imaging studies (e.g., Calvert et al., 2000, 2001; Beauchamp et al., 2004; Noesselt et al., 2007; Stevenson et al., 2007; Vander Wyk et al., 2010).

Importantly, we found that temporal congruence significantly modulated the functional connectivity between regions within the temporal, parietal and frontal lobes. We showed that there was an increase in functional connectivity between functionally localized auditory seed regions in the temporal lobe and frontal target regions when the auditory and visual signals had incongruent relative to congruent modulation rates. We also found that a functionally localized multi-sensory region in the right posterior STS showed increased functional connectivity with both parietal and frontal target regions for temporally incongruent as opposed to congruent AV stimuli. Lastly, we found a marginally significant increase in functional connectivity between the auditory seed region within the right STG and a target region within the left STG with congruent compared to incongruent AV stimuli. Our connectivity results are consistent with previous work showing inter-regional interactions during AV integration across a variety of stimuli and tasks (e.g., Noesselt et al., 2007, 2012; Lewis and Noppeney, 2010; Noppeney et al., 2010; Werner and Noppeney, 2010; Lee and Noppeney, 2011; Ogawa and Macaluso, 2013; Kim et al., 2015).

We found regional interactions predominantly between bilateral regions within the anterior STS and regions within the right frontal gyrus including inferior, middle, superior and medial regions for temporally incongruent AV stimuli (see **Table 5**; Bushara et al., 2001; Dhamala et al., 2007; Noesselt et al., 2012). Noesselt et al. (2012) recently reported greater functional connectivity between the STS and frontal regions when observers perceived AV stimuli to be asynchronous (i.e., temporally incongruent) relative to when they perceived the AV stimuli to be synchronous even though the stimuli were always physically asynchronous. In their study, Noesselt et al. (2012) used dynamic faces and voices and adjusted the stimulus onset asynchrony of facial movements and voices to produce temporally bistable percepts. They suggested that asynchronous perception is more demanding than synchronous perception as it requires the maintenance of two separate working memory representations (i.e., the auditory and visual percepts); hence the increased functional connectivity with the prefrontal cortex. In Noesselt et al.’s (2012) study, the functional connectivity was between multi-sensory regions within more posterior STS and prefrontal regions. We found that auditory regions in more anterior STS and a multi-sensory region in the posterior STS both showed increased functional connectivity with frontal regions, thereby demonstrating a large network of temporal and frontal regions (among others) in supporting AV integration. Our results further help generalize Noesselt et al.’s (2012) findings to non-ambiguous perception. The non-ambiguous nature of our stimuli may have led to the increased functional connectivity between auditory regions in the STS and frontal regions.

Noppeney et al. (2010) proposed another role for regional interactions between the STS and frontal regions. In their study, Noppeney et al. manipulated the reliability of auditory and visual information. Participants judged whether a stimulus was a tool or a musical instrument in eight different conditions derived by manipulating whether the auditory signal was intact or degraded (thereby reducing its reliability), whether the visual signal was intact or degraded, and whether the auditory and visual signals were congruent (i.e., same category) or incongruent (i.e., different categories). The authors found that the inferior frontal sulcus (IFS) inhibited superior temporal activations for unreliable auditory input, and suggested that the IFS accumulates AV evidence by weighting its connectivity to auditory or visual cortex according to the stimulus reliability and the salience of each modality for a perceptual decision. Other researchers have proposed that the STS and frontal regions may form a network that combines sensory and semantic information and that premotor cortex in the frontal lobe may be particularly important for integrating auditory and visual information for speech and other body movements (e.g., Meyer et al., 2011; Wuerger et al., 2012). However, these latter studies did not measure connectivity between these regions.

Lastly, we found that temporal congruence did not modulate the functional connectivity between visual seed regions and any other brain regions. This modulation may not have occurred for visual regions because vision tends to be a more reliable source of sensory information than audition (Witten and Knudsen, 2005). However, in future work, it would be interesting to systematically degrade the reliability of the auditory or visual signal. With our stimuli, we can reduce the magnitude of the modulations which may be a more naturalistic method of degradation than adding noise (e.g., Stevenson et al., 2007; Stevenson and James, 2009; Noppeney et al., 2010).

Interestingly, there is evidence that frontal regions may be more involved in integrating AV communication signals (e.g., Sugihara et al., 2006) or semantic categorization (e.g., Meyer et al., 2011; Wuerger et al., 2012). Vander Wyk et al. (2010) also showed that an ellipse combined with congruent speech led to activations in frontal regions whereas a circle combined with congruent speech did not. The authors argued that the ellipse was mouth-like and therefore resembled lips more than the circle did. Further work is needed to investigate the extent to which activation in frontal regions to AV stimuli and their functional connectivity with other regions are driven by stimulus properties (e.g., familiarity or duration) as opposed to task demands and attention. Our stimuli and paradigm could be systematically manipulated (e.g., reducing the stimulus duration) to address this question (see also Vander Wyk et al., 2010).

There are two outstanding issues that we did not address in the current study. First, PPI analyses do not indicate the direction of connectivity. Future work is needed to determine whether auditory and visual information is transmitted in a bottom–up stimulus-driven manner from uni-sensory to multi-sensory and frontal regions or whether there is top–down feedback from higher to lower regions, for example, using dynamic causal modeling (e.g., Lewis and Noppeney, 2010; Werner and Noppeney, 2010; Lee and Noppeney, 2011; Ogawa and Macaluso, 2013). Second, the functional connectivity between regions within the STS and the frontal lobe may reflect neural inhibition rather than AV integration. That is, the frontal regions may help to reduce responses to the incongruent signal in the unattended modality. However, the results of Noppeney et al. (2010) and Noesselt et al. (2012) suggest that our findings are due to AV integration (although we cannot completely rule out neural inhibition).

One advantage of our stimuli is that they capture key aspects of naturalistic stimuli such as speech yet do not carry any semantic content (see also Vander Wyk et al., 2010). We are also able to manipulate the auditory and visual signals in comparable ways (i.e., modulation of the amplitude or size). With our current stimuli, there is some degree of correlation even when the auditory and visual signals have different modulation rates because the “fast” modulation rate (2 Hz) is a harmonic of the “slow” one (1 Hz) and close in value (see **Figure 1**). However, in a separate study using these stimuli, we found that the AV congruent stimulus affected performance on an amplitude-modulation discrimination task, but not the AVI stimuli (Vuong et al., 2014). This finding suggests that observers’ were sensitive to the difference in temporal congruence between the two types of AV stimuli. It would be interesting in future work to more systematically manipulate the frequency difference and the harmonicity between the modulation rates.

## Conclusion

In summary, using amplitude-modulated tones and size-modulated shapes, our functional imaging study revealed the importance of both regional activation and inter-regional connectivity in AV integration across a network of temporal, parietal, and frontal regions. Supporting our findings, diffusion imaging data in humans suggest that there are anatomical connections between some of these regions (Beer et al., 2011, 2013; van den Brink et al., 2014). Moreover, recent studies in non-human primates suggest that there are also effective functional (Petkov et al., 2015) and anatomical (Yeterian et al., 2012) connections between the STS and frontal regions. Compared to congruent stimuli, temporally incongruent stimuli elicited increased functional connectivity between auditory and multi-sensory regions in the STS and prefrontal regions. Importantly, these physiological changes were obtained using continuously varying non-meaningful stimuli. The AV interactions observed in this study are not confounded by semantic content, and therefore they provide an important link between transient, non-meaningful stimuli and continuous real-world objects, speech and music.

## Conflict of Interest Statement

The authors declare that the research was conducted in the absence of any commercial or financial relationships that could be construed as a potential conflict of interest.

## Footnotes

1 http://www.psychtoolbox.org

2 http://www.fil.ion.ucl.ac.uk/spm

3 www.nitrc.org/projects/gppi
